# Using hexamers to predict cis-regulatory motifs in Drosophila

**DOI:** 10.1186/1471-2105-6-262

**Published:** 2005-10-27

**Authors:** Bob Y Chan, Dennis Kibler

**Affiliations:** 1 School of Information and Computer Science, University of California, Irvine, Irvine, California, USA

## Abstract

**Background:**

Cis-regulatory modules (CRMs) are short stretches of DNA that help regulate gene expression in higher eukaryotes. They have been found up to 1 megabase away from the genes they regulate and can be located upstream, downstream, and even within their target genes. Due to the difficulty of finding CRMs using biological and computational techniques, even well-studied regulatory systems may contain CRMs that have not yet been discovered.

**Results:**

We present a simple, efficient method (HexDiff) based only on hexamer frequencies of known CRMs and non-CRM sequence to predict novel CRMs in regulatory systems. On a data set of 16 gap and pair-rule genes containing 52 known CRMs, predictions made by HexDiff had a higher correlation with the known CRMs than several existing CRM prediction algorithms: Ahab, Cluster Buster, MSCAN, MCAST, and LWF. After combining the results of the different algorithms, 10 putative CRMs were identified and are strong candidates for future study. The hexamers used by HexDiff to distinguish between CRMs and non-CRM sequence were also analyzed and were shown to be enriched in regulatory elements.

**Conclusion:**

HexDiff provides an efficient and effective means for finding new CRMs based on known CRMs, rather than known binding sites.

## Background

The development of eukaryotic organisms is tightly regulated by a variety of mechanisms. The initial step of regulation is carried out by transcription factors interacting with cis-regulatory sequences, also known as transcription factor binding sites (TFBS). In eukaryotes, multiple TFBS are often clustered together into cis-regulatory modules (CRMs). The TFBS can be thought of as inputs into an information processing element, with the output being the level of expression of the gene controlled by the CRM [1].

One of the major challenges for understanding eukaryotic gene regulation is finding CRMs. There are two main types of CRMs – promoters and enhancers. Promoters are located immediately upstream of a gene's transcriptional start site and often contain a variety of sequence signals such as the TATA box, CCAAT box, and different TFBS. These characteristics have been used in approaches for finding promoters [2]. In contrast, enhancers do not share these signals and operate in a manner that is relatively independent of orientation or distance from their target gene [3]. In fact, one enhancer, Dct, has been found almost a megabase away from Sox9, the gene it regulates [4]. Because of the lack of common signals and because the search for enhancers cannot be limited to the few hundred base pairs upstream of the transcriptional start site, finding enhancers is a more difficult problem.

Methods for predicting CRMs can be classified by the type of information they use to make the predictions – known binding sites of regulatory proteins, homologous sequences, or known CRMs. Binding sites for the first type of method are generally modeled using position weight matrices (PWMs) or consensus sequences. These models are used to search for statistically significant clusters of predicted TFBS. Examples of methods based on binding sites of multiple transcription factor proteins include one developed for human skeletal muscle [5], a logistic regression analysis model for liver-specific transcription factors [6], CIS-ANALYST [7], MCAST [8], Ahab [9], Stubb [10], Cluster Buster [11], MSCAN [12], and EMCMODULE [13]. Methods based on binding sites of single transcription factors have also been developed – SCORE [14], Fly Enhancer [15], and a method of searching for homotypic clusters [16].

Methods based on homologous sequences assume that areas of the DNA involved in regulating gene transcription are under selective pressure and are therefore more likely to be conserved than non-functional DNA [17]. These methods can be categorized by whether they search for conserved DNA by aligning homologous regions from multiple species [18-20], homologous regions from two species [21-24], or homologous regions from related genes in a single species (also referred to as co-regulated genes) [25-27].

Methods based on locations of known CRMs have been rarer. Methods of this type tend to look for statistical properties of DNA sequence that distinguish CRMs from non-regulatory DNA. One group has developed a statistical test called the "fluffy-tail test" that looks for differences in nucleotide composition, particularly in lists of words of various lengths [28]. From the related field of promoter prediction comes PromFind, an algorithm for finding promoters that uses hexamer frequencies of known promoters to search for DNA with similar frequencies [29]. Because PromFind was developed for promoters, the author could assume that every sequence being tested contained one promoter, and that the strand containing the promoter was known – assumptions that are not true for enhancers. Another recent algorithm developed to predict CRMs is based on the exhaustive analysis of local word frequencies (LWF) [30]. Unlike PromFind where the algorithm is based solely on hexamer words, the LWF algorithm considers the pattern of word frequencies in a sliding window.

While the LWF algorithm was shown to perform well at the task of predicting CRMs [30], it is difficult to put a biological meaning to the results since the algorithm depends on word frequencies, not on the words themselves. In contrast, the PromFind algorithm generates lists of hexamers that are important for distinguishing promoters from non-promoter sequences. In the paper describing PromFind, the author analyzes the hexamers used by the algorithm for their CpG dinucleotide content and their similarity to various known promoter signals such as the Sp1, TATA-box, and CCAAT-box motifs [29]. This type of analysis cannot be performed on the local word frequency distributions of the LWF algorithm, due to the way it was designed.

We developed the HexDiff algorithm to solve the same problem as the LWF algorithm – predicting the location of CRMs – while being as biologically meaningful as the PromFind algorithm. The performance of HexDiff was compared to the LWF algorithm and several other CRM prediction programs using a common data set. The hexamers used by HexDiff were then examined to see if known TFBS were recovered.

## Results

### Data set

The early development of the Drosophila embryo is well-studied, both biologically and computationally. Data sets detailing the locations of known CRMs have been published by several groups [7,9,30,31], but we chose the one compiled by Schroeder *et al*. as it clearly defined the regulatory networks involved. In their analysis, Schroeder *et al*. examined 29 genes with gap and pair-rule patterns. However, only 16 of those genes were associated with known CRMs [31]. Therefore, we focused our study on those 16 genes, which contained a total of 52 CRMs. Sequences for the genes were obtained from the 4.1 Release of the Drosophila genome and included the 20 kb upstream as well as downstream of each gene [see Additional file 1]. Since the known CRMs were provided as FASTA-formatted DNA sequences, their positions relative to the extracted sequences were confirmed using BLAST [see Additional file 2].

### The HexDiff algorithm

The HexDiff algorithm is designed to discriminate between CRMs and non-CRM sequence by using hexamer frequencies. For evaluation, we use the leave-one-out cross-validation (LOOCV) methodology. In this case, 15 of the 16 sequences in the data set are used as a training set and the 16^th ^sequence is used as the test set. The process is repeated 16 times; leaving out one sequence each time. During training, hexamers that appear more frequently in CRMs are selected. In order to predict CRMs in the test set sequence, a window is slid across the sequence and the set of selected hexamers (H_d_) is used to calculate a score that is used to predict whether each position is either CRM or non-CRM sequence.

### Performance comparison

The HexDiff algorithm was compared to five other CRM prediction algorithms: Ahab, Cluster Buster, MSCAN, MCAST, and LWF (see Table 1). The results for Ahab were obtained from the supplementary files of Schroeder *et al*., while Cluster Buster, MSCAN, MCAST, and LWF are all available as downloadable software or public web servers. An important criterion for the inclusion of an algorithm was that it accepted user-defined sequences and PWMs as input. Ahab, Cluster Buster, MSCAN, and MCAST are algorithms based on binding sites of known transcription factors and were given the same set of nine PWMs for the transcription factors as described in Schroeder *et al*.: the maternal factors Bicoid (Bcd), Hunchback (Hb), Caudal (Cad), the Torso-response element (TorRE), and Stat92E (D-Stat), and the gap factors Kruppel (Kr), Knirps (Kni), Giant (Gt), and Tailless (Tll). LWF was given the same positive and negative training sets as HexDiff. The default parameters were used for Cluster Buster, MSCAN, MCAST, and LWF. The complete list of predictions can be found in the Additional materials section [see Additional file 3].

**Table 1:** Key aspects of HexDiff and other algorithms. The table shows the knowledge used and the parameters required by the different algorithms.

Algorithm	Knowledge Used	Parameters
HexDiff	CRM Locations	Number of hexamers in H_d_ Window size Window score threshold
Ahab	PWMs	Window size Free energy cutoff Order of background model
Cluster Buster	PWMs	Motif score threshold Gap parameter Cluster score threshold Residue abundance range
MSCAN	PWMs	Motif score threshold Window size Minimum hits Maximum hits
MCAST	PWMs	Motif score threshold Maximum allowed distance between adjacent hits Pseudocount weight
LWF	CRM Locations	String length Number of mismatches Detection window size Maximum number of channels Channels equalized Profile cutoff Peak width cutoff Smoothing window

As shown in Table 2, each algorithm was assigned a cumulative score, calculated by summing the Matthews correlation coefficients for each of the sequences in the data set. The predictions made by HexDiff have the highest correlation with the known CRMs (5.71), followed by Ahab (4.48) and Cluster Buster (4.24). An interesting result is that although four of the six algorithms used the same PWMs, their performance varied widely – from 1.81 to 4.48.

**Table 2:** Correlation between predicted and known CRMs. The performance of six different algorithms on a common data set is compared in this table. For each sequence, the Matthews correlation coefficient is calculated by checking whether each position is a TP, TN, FP, or FN and using the equation listed in the Methods section. The sum of the correlation coefficients gives a cumulative score for each algorithm on this data set.

Gene	CRMs	HexDiff	Ahab	Cluster Buster	MSCAN	MCAST	LWF
btd	1	0.70	0.57	0.19	0.01	0.07	0.10
ems	3	0.00	0.00	-0.03	0.12	-0.01	-0.01
eve	6	0.55	0.63	0.65	0.50	0.41	0.06
fkh	1	-0.03	-0.02	-0.02	-0.04	-0.02	-0.01
ftz	5	0.40	0.28	0.28	0.07	0.16	0.08
gt	1	0.27	0.42	0.33	0.35	0.15	0.03
h	5	0.71	0.63	0.53	0.30	0.37	0.08
hb	2	0.35	0.63	0.39	0.34	0.24	0.04
hkb	1	0.51	0.00	-0.02	-0.02	-0.08	0.09
kni	3	0.55	0.55	0.39	0.37	0.23	-0.05
kr	3	0.43	0.00	0.77	0.20	0.11	-0.03
oc	2	0.70	-0.02	0.00	0.11	0.02	0.07
prd	7	0.01	-0.07	0.16	0.07	-0.04	0.05
run	6	0.27	0.16	0.08	0.08	0.02	0.07
slp1	3	-0.07	0.15	-0.04	0.00	0.07	0.01
tll	3	0.35	0.56	0.58	0.19	0.12	-0.04
Total	52	5.71	4.48	4.24	2.64	1.81	0.52

While the Matthews correlation coefficient was the primary performance measure for the six algorithms, a closer look at two other measures offers more information about the characteristics of the individual algorithms. Table 3 shows sensitivities and positive predictive values (PPVs) for each of the algorithms. A known CRM was considered recovered if the overlap between it and a predicted CRM exceeded 50 bp.

**Table 3:** Sensitivities and positive predictive values (PPVs) of HexDiff and other algorithms. A known CRM was considered recovered if a predicted CRM overlapped it by at least 50 bp. The PPVs in this table are italicized because they are estimates of the true PPVs. Without complete knowledge of all CRMs that are present in the 16 sequences, it is possible that some of the predicted CRMs that are labeled as false positives are actually true positives.

Algorithm	CRMs Recovered	Num CRMs	Sensitivity TP/(TP + FN)	True Positives	CRMs Predicted	PPV TP/(TP + FP)
HexDiff	36	52	69.23%	35	104	*33.65%*
Ahab	23	52	44.23%	20	35	*57.14%*
Cluster Buster	31	52	59.62%	23	88	*26.14%*
MSCAN	34	52	65.38%	42	226	*18.58%*
MCAST	43	52	82.69%	53	499	*10.62%*
LWF	27	52	51.92%	48	433	*11.09%*

One caveat about the PPVs in Table 3 is that they are not true PPVs, but estimates of the true PPVs. This is due to the fact that the 16 sequences in the data set may contain more CRMs than the 52 that have been characterized so far, which would mean that some of the predicted CRMs that are labeled as false positives could actually be true positives.

### Predicted CRMs

Of the 104 predictions made by HexDiff, 36 overlapped known CRMs by at least 50 bp, leaving 68 potential CRMs. Some of these may have been false positives, so to narrow down the candidates, the 68 potential CRMs were compared to predictions made by Ahab, Cluster Buster, MSCAN, MCAST, and LWF. 37 of the 68 matched predictions made by at least one other method, while 18 matched predictions made by at least two other methods, 10 matched predictions made by at least three other methods, and 6 matched predictions made by at least four other methods.

While our analysis was focused on the gap and pair-rule regulatory networks, it's possible that some of the predicted CRMs are actually known CRMs from other regulatory networks involved in the early development of Drosophila. Therefore, the list of 18 predicted CRMs was compared to a more comprehensive compilation of 124 CRMs [32]. 8 of the 18 predicted CRMs matched CRMs from the compilation, leaving 10 that do not correspond to any known CRMs. Given the specificities of the individual methods and the breadth of approaches, it seems very likely that some of the 18 predictions listed in Table 4 constitute novel CRMs.

**Table 4:** Potential novel CRMs predicted by HexDiff and other algorithms. All of the predicted CRMs listed in this table were predicted by HexDiff and at least two other algorithms. The column labeled "Gene" lists the gene involved in the early development of Drosophila that is closest to the predicted CRM. The columns labeled 1–5 are the different algorithms whose predictions matched the CRMs predicted by HexDiff: 1 – Ahab, 2 – Cluster Buster, 3 – MSCAN, 4 – MCAST, and 5 – LWF. The predicted CRMs were also compared to a compilation of 124 CRMs [32] – matching CRMs are listed in the last column.

Gene	Arm	Begin	End	Length	1	2	3	4	5	Matched
btd	X	9534921	9535192	271				*	*	
eve	2R	5492385	5493575	1190				*	*	eve_late2_mel
fkh	3R	24421705	24422385	680				*	*	
ftz	3R	2683060	2683406	346			*	*		
gt	X	2268347	2270179	1832		*	*	*		
gt	X	2290228	2290685	457	*	*	*	*	*	gt_23-bcd_mel
hb	3R	4503375	4503962	587			*	*	*	
hb	3R	4519805	4520172	367			*	*		
kni	3L	20628230	20628504	274	*	*	*	*		kni_+1_mel
prd	2L	12080435	12082316	1881	*			*	*	prd_bcd_mel
prd	2L	12089627	12089847	220				*	*	prd_1_mel
run	X	20488169	20488643	474	*	*	*	*		
run	X	20524260	20524722	462		*	*	*	*	
slp1	2L	3811050	3812092	1042				*	*	
slp1	2L	3822581	3823049	468				*	*	
slp1	2L	3824891	3825039	148	*	*	*	*	*	slp_A-bcd_mel
slp1	2L	3833433	3834671	1238		*	*	*	*	slp2_-3_mel
tll	3R	26680559	26683175	2616		*		*	*	tll_bcd_mel

### Meaning of differential hexamers

One advantage of the HexDiff algorithm is that the set of hexamers (H_d_) used to distinguish between CRMs and non-CRM sequence can be analyzed for further insights into the mechanisms of gene regulation. Since the H_d _hexamers were selected based on their overrepresentation in CRMs relative to non-CRM sequence, it would be reasonable to expect that some of the hexamers would be similar to gap and pair-rule regulatory sites. Therefore, the top 80 H_d _hexamers and their reverse complements were compared to the list of binding sites used to build the 9 PWMs from Schroeder *et al *[see Additional file 4]. In the 16 rounds of cross-validation, an average of 59.6 hexamers was found within the binding sites.

Simulations were carried out to estimate the likelihood of this result. 100,000 random sets of 80 hexamers and their reverse complements were compared to the binding sites. The probability of 59 or more hexamers being found within the binding sites was 0.019, indicating that the hexamers selected by HexDiff were enriched in binding sites of known regulatory proteins.

A similar study was performed using regulatory sites obtained from TRANSFAC [33], but the results were not statistically significant. TRANSFAC contains binding regions obtained through a variety of biological methods. For instance, R02491 contains the following binding region obtained using DNase I footprinting: GACTTTATTGCAGCATCTTGAACAATCGTCGCAGTTTGGTAACAC. On average, TRANSFAC binding sites are much longer than the binding sites used by Schroeder *et al *.and are therefore probably much less specific. While the TRANSFAC binding sites do contain regulatory DNA, it seems that the signal is washed out by extraneous sequence.

## Discussion

The HexDiff algorithm is designed to solve the difficult task of distinguishing CRMs from non-CRM sequence. It is first trained on sequences containing known CRMs by selecting hexamers that discriminate between the two categories. These hexamers are then applied to novel sequences to search for predicted CRMs. The requirements of the training process mean that HexDiff works best in a well-defined regulatory system where some CRMs are already known.

Using a data set of 16 sequences containing 52 CRMs obtained from Schroeder *et al*., we compared the HexDiff algorithm to five other algorithms: Ahab, Cluster Buster, MSCAN, MCAST, and LWF. Hexdiff's predictions correlated best with biological knowledge, and its sensitivity and specificity were comparable to the other algorithms. This result was encouraging considering that HexDiff is a type of machine learning algorithm, which tend to do better in problems where the items being classified are separated into two roughly equal groups. In this case, the CRMs made up just 9.36% of the total data set, with non-CRM sequence making up the other 90.64%. Even with a data set where the negative data outweighed the positive data by a factor of 9, HexDiff still performed well.

While the HexDiff algorithm has a fairly high specificity in isolation, it would still be prudent to compare its results to the predictions made by other algorithms, considering the time and effort required to biologically confirm a computational prediction. The 18 CRMs listed in Table 4 were predicted by at least three algorithms and did not match any of the 52 CRMs provided by Schroeder *et al*. A comparison with a more comprehensive list of 124 CRMs revealed that 8 of the 18 predicted CRMs corresponded to known CRMs from other regulatory networks involved in the early development of Drosophila. The 10 remaining predicted CRMs are strong candidates for future study.

A further attempt to understand the meaning of the H_d _hexamers was made by comparing them to the known binding sites used to generate the 9 PWMs provided by Schroeder *et al*. Simulations showed that the number of H_d _hexamers found within the binding sites was significantly more than would be expected of a randomly selected set of hexamers of the same size. While this result is not unexpected, it is a confirmation that we retrieved the binding sites of relevant regulatory proteins using only the locations of known CRMs.

While recovering the known binding sites is important, it is important to note that they accounted for less than half of the hexamers in the H_d _sets. When 80 hexamers were selected for H_d_, their reverse complements would increase the total number of hexamers to 160, barring palindromes. And yet, the average number of hexamers that were found within the known binding sites was 59.6, leaving another 100 hexamers whose identities are unknown. This result suggests that there are novel sequence features besides the known binding sites that are important for distinguishing between CRMs and non-CRM sequence.

## Conclusion

One of the major questions in studying eukaryotic gene regulation is how regulatory proteins with relatively degenerate binding sequences can precisely regulate many genes. The discovery of cis-regulatory modules, short stretches of DNA that contain multiple binding sites for multiple proteins, has provided at least a partial explanation for the regulatory specificity observed in eukaryotes, and motivated a search for ways to predict CRMs computationally.

We have developed a simple and effective algorithm for predicting CRMs. In our study of the gap and pair-rule genes in *Drosophila melanogaster*, the results of the HexDiff algorithm correlated best with biological knowledge, and the sensitivity and specificity of the algorithm were comparable to other algorithms. Our predictions were compared to those made by other methods and resulted in a list of 10 putative CRMs with strong computational support. Analysis of the H_d _hexamers revealed that not only were we rediscovering the known binding sites, but also discovering new signals that distinguished between CRMs and non-CRM sequence.

## Methods

### Differential hexamer frequency algorithm

The HexDiff algorithm is based on the idea of distinguishing between two types of DNA sequence: CRMs, and non-CRM sequence. In order to accomplish this task, a model is built using sequences where the CRMs are known – the training set. The training set is split into positive and negative training sets by consolidating all of the known CRMs into a positive training set, and the remainder of the training set into a negative training set. On average, the ~1 Mb training set is split into a ~50 kb positive training set and a ~950 kb negative training set. The frequency of each hexamer *h *on both strands is then calculated for the positive *f*_*p*_*(h) *and negative *f*_*n*_*(h) *training sets and used to calculate a ratio, *R(h)*.

R(h)=fp(h)fn(h)
 MathType@MTEF@5@5@+=feaafiart1ev1aaatCvAUfKttLearuWrP9MDH5MBPbIqV92AaeXatLxBI9gBaebbnrfifHhDYfgasaacH8akY=wiFfYdH8Gipec8Eeeu0xXdbba9frFj0=OqFfea0dXdd9vqai=hGuQ8kuc9pgc9s8qqaq=dirpe0xb9q8qiLsFr0=vr0=vr0dc8meaabaqaciaacaGaaeqabaqabeGadaaakeaacqWGsbGucqGGOaakcqWGObaAcqGGPaqkcqGH9aqpdaWcaaqaaiabdAgaMnaaBaaaleaacqWGWbaCaeqaaOGaeiikaGIaemiAaGMaeiykaKcabaGaemOzay2aaSbaaSqaaiabd6gaUbqabaGccqGGOaakcqWGObaAcqGGPaqkaaaaaa@3DF4@

The next step is to select hexamers that discriminate between CRMs and non-CRM sequence. The hexamers with the highest values for *R(h) *are then placed into H_d_. As a result of this selection process, H_d _contains hexamers that are much more common in CRMs than in non-CRM sequence.

Once the hexamers for H_d _are chosen, the final step is to use them to classify each position in an unknown sequence as either CRM or non-CRM sequence. A window is slid across the unknown sequence 1 bp at a time. At each position *i*, a score *S*_*i *_is calculated for the window by increasing the score by the product of *R*(*h*_*d*_) and the number of times that hexamer appeared *n*(*h*_*d*_) for each hexamer from H_d_:

Si=∑hd∈Hd[n(hd)R(hd)]
 MathType@MTEF@5@5@+=feaafiart1ev1aaatCvAUfKttLearuWrP9MDH5MBPbIqV92AaeXatLxBI9gBaebbnrfifHhDYfgasaacH8akY=wiFfYdH8Gipec8Eeeu0xXdbba9frFj0=OqFfea0dXdd9vqai=hGuQ8kuc9pgc9s8qqaq=dirpe0xb9q8qiLsFr0=vr0=vr0dc8meaabaqaciaacaGaaeqabaqabeGadaaakeaacqWGtbWudaWgaaWcbaGaemyAaKgabeaakiabg2da9maaqafabaGaei4waSLaemOBa4MaeiikaGIaemiAaG2aaSbaaSqaaiabdsgaKbqabaGccqGGPaqkcqWGsbGucqGGOaakcqWGObaAdaWgaaWcbaGaemizaqgabeaakiabcMcaPiabc2faDbWcbaGaemiAaG2aaSbaaWqaaiabdsgaKbqabaWccqGHiiIZcqWGibasdaWgaaadbaGaemizaqgabeaaaSqab0GaeyyeIuoaaaa@47D3@

Any positions where the window scores exceed a threshold score are labeled as potential CRMs. As the shortest known CRM in the data set was 66 bp, we filtered out any predicted CRMs shorter than 50 bp. The entire process takes less than 10 seconds per gene on an Athlon 64 3200+.

### Evaluation of predictions

Because there are only 16 sequences in the data set, LOOCV was used to assess the performance of the HexDiff algorithm. In LOOCV, the first of the 16 sequences was withheld from the data set and used as the test set. The HexDiff algorithm was trained on the 15 remaining sequences and predictions were made on the test set sequence. Those predictions were then compared to the locations of the known CRMs in the test set sequence. The same process was repeated with the second sequence, etc., until each of the 16 sequences had been used as a test set.

The accuracy of the predictions for each sequence was measured using the Matthews correlation coefficient, which has been used extensively to evaluate the performance of various prediction algorithms [34]. It combines both sensitivity and specificity into one measure and relies on four values that satisfy TP + TN + FP + FN = N (length of the sequence): TP (the number of base pairs where known CRMs overlap with predicted CRMs), TN (the number of base pairs that are not in known CRMs or predicted CRMs), FP (the number of base pairs where predicted CRMs did not overlap known CRMs), and FN (the number of base pairs where known CRMs did not overlap predicted CRMs). The Matthews correlation coefficient is calculated as follows:

CC=TP*TN−FP*FN(TP+FP)(TP+FN)(TN+FP)(TN+FN)
 MathType@MTEF@5@5@+=feaafiart1ev1aaatCvAUfKttLearuWrP9MDH5MBPbIqV92AaeXatLxBI9gBaebbnrfifHhDYfgasaacH8akY=wiFfYdH8Gipec8Eeeu0xXdbba9frFj0=OqFfea0dXdd9vqai=hGuQ8kuc9pgc9s8qqaq=dirpe0xb9q8qiLsFr0=vr0=vr0dc8meaabaqaciaacaGaaeqabaqabeGadaaakeaacqWGdbWqcqWGdbWqcqGH9aqpdaWcaaqaaiabdsfaujabdcfaqjabcQcaQiabdsfaujabd6eaojabgkHiTiabdAeagjabdcfaqjabcQcaQiabdAeagjabd6eaobqaamaakaaabaGaeiikaGIaemivaqLaemiuaaLaey4kaSIaemOrayKaemiuaaLaeiykaKIaeiikaGIaemivaqLaemiuaaLaey4kaSIaemOrayKaemOta4KaeiykaKIaeiikaGIaemivaqLaemOta4Kaey4kaSIaemOrayKaemiuaaLaeiykaKIaeiikaGIaemivaqLaemOta4Kaey4kaSIaemOrayKaemOta4KaeiykaKcaleqaaaaaaaa@5868@

The Matthews correlation coefficient ranges from -1 to +1, like the better-known Pearson correlation coefficient. A value of 0 signifies that the prediction is equivalent to a completely random prediction, while +1 signifies a perfect prediction.

### Choosing parameters

The HexDiff algorithm was designed so that the number of parameters needed to be set by the user was minimized. This reduced the complexity of the model and helped to avoid overfitting. Overfitting is a problem often faced by machine learning algorithms, where a model that is too complex will not only learn the signal in the training set but will also fit the noise, reducing the algorithm's performance on the test set [35]. The three parameters HexDiff does require are: the number of hexamers in H_d_, the size of the window that is slid across the sequence of interest, and the threshold score that determines whether each position is predicted as a CRM or not.

These values were chosen during LOOCV using an empirical method. During each round of cross-validation, the 16 sequences were split into a 15-sequence training set and a single-sequence test set. The HexDiff algorithm was trained on the training set and its performance evaluated on the training set, using various combinations of the three parameters. The combination that gave the best performance on the training set was then used to predict CRMs in the test set.

In general, we observed that the HexDiff algorithm was relatively insensitive to the precise values for the parameters. We ran the HexDiff algorithms with 6 values for the number of hexamers (30, 40, 50, 60, 70, 80) and 11 values for the size of the sliding window (1000, 1100, 1200, 1300, 1400, 1500, 1600, 1700, 1800, 1900, 2000). For each pair of parameters, the threshold score that gave the best performance on the training set was selected. For these 66 analyses, the average cumulative Matthews correlation coefficient for the test set sequences was 5.37 with a standard deviation of 0.51. The average number of modules recovered was 33.8 with a standard deviation of 3.29.

### Choices in algorithm design

Two important choices were made during the design of the HexDiff algorithm: the model used to represent the training sets, and the type of negative training set used. In order to choose the correct model, a balance had to be struck between the expressivity of the model and the amount of training data. Since the amount of positive training data was fixed at 52 known CRMs, we tried pentamers, hexamers, and heptamers with 0, 1, or 2 mismatches (data not shown). Shorter n-mers would have resulted in a model that was insufficiently expressive to capture the difference between CRMs and non-CRM sequences, while longer n-mers would have resulted in a model whose parameters would have had high variance. Allowing mismatches would have consolidated n-mers into groups and therefore would have had the effect of simplifying the model. In the end, hexamers with no mismatches turned out to have the best performance.

For the LWF algorithm, Nazina and Papatsenko tried three different negative training sets drawn from the Drosophila genome: random samples from the whole genome, random samples of coding sequence, and random samples of non-coding sequence. They found that samples from the whole genome and samples from non-coding sequence resulted in better agreement between CRM predictions from the LWF algorithm and the biologically determined locations. Since we were predicting CRMs in a well-defined set of 16 genes, we used a local negative training set – the portions of the 16 genes that were not labeled as known CRMs. We found this approach to give higher accuracy than the negative training sets used by Nazina and Papatsenko (data not shown).

## Availability and requirements

Project name: HexDiffProject home page: 

Operating system: Platform independent

Programming language: Perl

Other requirements: Perl 5.6 or higher

License: GNU GPL

Any restrictions to use by non-academics: licence needed

## Authors' contributions

BC carried out the computational work in this study and drafted the manuscript. DK participated in the design of the study and helped revise the manuscript. Both authors read and approved the final manuscript.

## Supplementary Material

Additional File 1 **Sequences comprising the data set**. FASTA-formatted sequences that include the 20 kb upstream and downstream of the 16 gap and pair-rule genes that were studied. Sequences were extracted from Release 4.1 of the Drosophila genome using FlyBase's "GBrowse" Genome Browser. Click here for file

Additional File 2 **Coordinates of known CRMs**. Coordinates of known CRMs, relative to the sequences in Additional file 2. Click here for file

Additional File 3 **Coordinates of CRMs predicted by the different algorithms**. Coordinates of predicted CRMs for each of the algorithms compared in this study, relative to the sequences in Additional file 2. Click here for file

Additional File 4 **Hd hexamers**. Top 80 H_d _hexamers calculated by the HexDiff algorithm for each round of cross-validation. Click here for file

Additional File 5 **ROC curves for the HexDiff algorithm**. The three curves in this plot were made by taking the combination of parameters that gave the best performance on the training sets (number of nmers: 80, window size: 1700 bp, threshold: 170) and holding two parameters constant while varying the third. Click here for file

Additional File 6 **Sensitivities and specificities for HexDiff and the other algorithms**. Sensitivities and specificities for HexDiff and the other algorithms were calculated by checking whether each position was a TP, FP, TN, or FN and using the appropriate formulas. Click here for file
