# Supplementary material for: Using hexamers to predict cis-regulatory motifs in Drosophila
Source: BMC Bioinformatics. 2005 Oct 27;6:262. doi: 10.1186/1471-2105-6-262 (PMC1291357; doi:10.1186/1471-2105-6-262)

Additional file 5

This graph was generated by taking the combination of parameters that gave the best performance (number of nmers: 80, window size: 1700 bp, threshold: 130) on the training set and holding two of the parameters constant, while varying the third. The number of nmers was varied between 30-80 in steps of 10, the window size was varied between 1000 and 2000 in steps of 100, and the threshold was varied between 0 and 200 in steps of 10. Note that varying the number of nmers without changing the other two parameters gave a maximum sensitivity of around 0.42. Varying the window size gave a maximum sensitivity of 0.68.

ROC curves were not prepared for the other algorithms as most of them had more parameters than HexDiff, making it difficult to summarize their performance into a practical number of ROC curves. For comparing the different algorithms, please refer to Tables 2 and 3 of the manuscript.

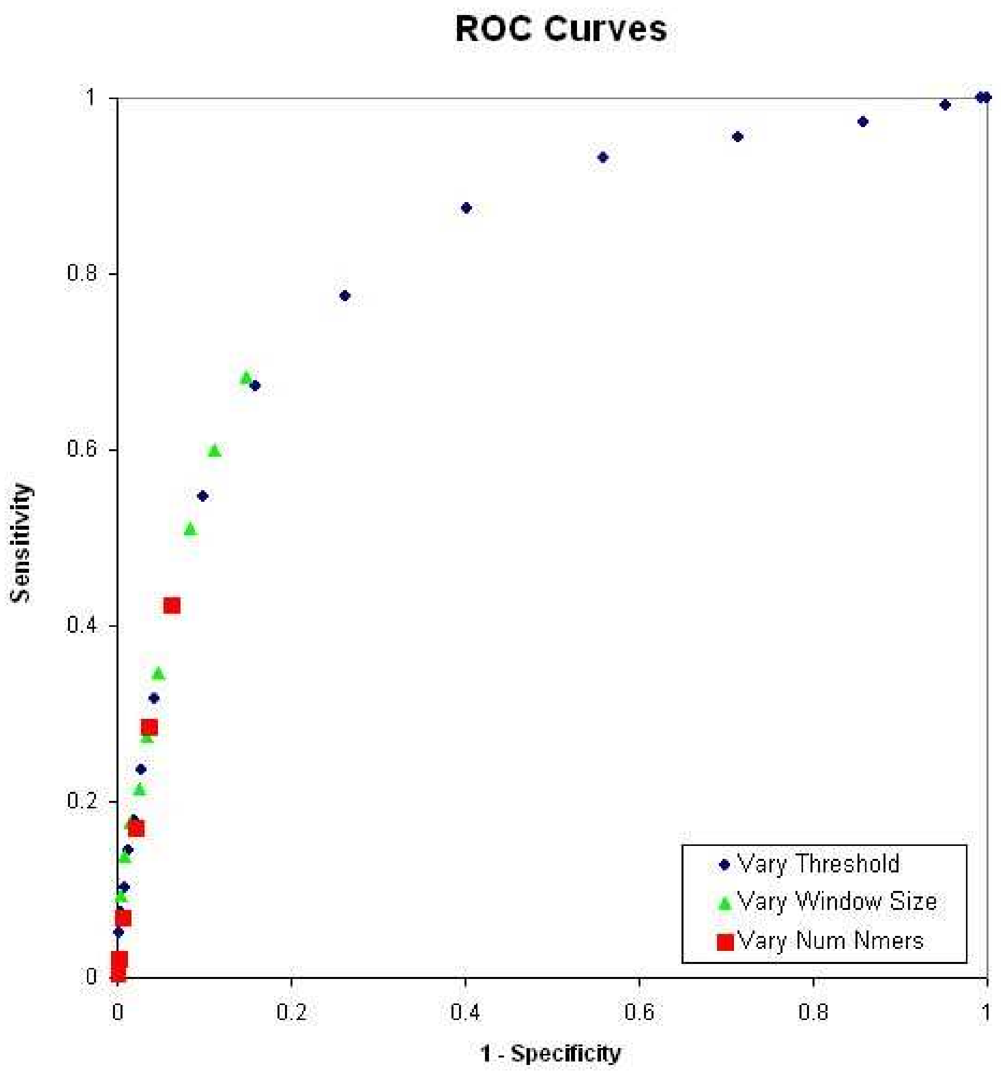

Supplement: Additional File 5 — ROC curves for the HexDiff algorithm. The three curves in this plot were made by taking the combination of parameters that gave the best performance on the training sets (number of nmers: 80, window size: 1700 bp, threshold: 170) and holding two parameters constant while varying the third. [file 1471-2105-6-262-S5.pdf]
