# Supplementary material for: Using hexamers to predict cis-regulatory motifs in Drosophila
Source: BMC Bioinformatics. 2005 Oct 27;6:262. doi: 10.1186/1471-2105-6-262 (PMC1291357; doi:10.1186/1471-2105-6-262)
Supplement: Additional File 6 — Sensitivities and specificities for HexDiff and the other algorithms. Sensitivities and specificities for HexDiff and the other algorithms were calculated by checking whether each position was a TP, FP, TN, or FN and using the appropriate formulas. [file 1471-2105-6-262-S6.pdf]

Additional file 6

The values for true positives (TP), false positives (FP), true negatives (TN), and false negatives (FN) were calculated by examining each position in the sequence, and checking whether it was part of a predicted CRM, part of a known CRM, both, or neither. These values were then used to calculate sensitivity, specificity, and positive predictive value (PPV).

	TP	FP	TN	FN	Sensitivity TP/(TP+FN)	Specificity TN/(TN+FP)	PPV TP/(TP+FP)
HexDiff	22548	40007	602501	35751	38.68%	93.77%	36.05%
Ahab	12862	10488	632020	45437	22.06%	98.37%	55.08%
Cluster Buster	19883	33339	609169	38416	34.11%	94.81%	37.36%
MSCAN	15771	58679	583829	42528	27.05%	90.87%	21.18%
MCAST	28009	194633	447875	30290	48.04%	69.71%	12.58%
LWF	7436	61165	581343	50863	12.75%	90.48%	10.84%
